# Role of liver biopsy in management of liver diseases without hepatic nodules following end of the interferon era: experience of a tertiary referral center

**DOI:** 10.1007/s10238-022-00797-1

**Published:** 2022-03-09

**Authors:** Nermine A. Ehsan, Maha M. Elsabaawy, Dina M. Sweed, Esraa A. Karman, Eman Abdelsameea, Anwar A. Mohamed

**Affiliations:** 1 Department of Pathology, National Liver Institute, Menoufia University, Shebeen El-Koom, Egypt; 2 Hepatology and Gasteroenterology, National Liver Institute, Menoufia University, Shebeen El-Koom, 32511 Egypt

**Keywords:** Liver biopsy, Liver diseases, Etiological diagnosis, Histopathological evaluation

## Abstract

Liver biopsy (LB) is the cornerstone in the management of patients with liver diseases. However, a lot of queries had emerged about its role following the end of the interferon era. The aim of this study was to re-evaluate the current role of LB in the diagnosis of liver diseases. All patients who had underwent LB at the Department of Hepatology, National Liver Institute, from January 2015 through December 2018 were recruited. Indications for LB, pathology reports and medical records of all cases were retrieved, reviewed and statistically analyzed. A total of 275 liver biopsies were collected, 191 males and 84 females with mean age 41.22 ± 13.36 years. Etiological diagnosis made by histopathological evaluation was 48 drug-induced liver injury (DILI), 42 nonalcoholic fatty liver disease (NAFLD), 34 chronic hepatitis B, or C with cholestasis, 29 autoimmune hepatitis, 34 primary sclerosing cholangitis, 13 primary biliary cholangitis, 7 autoimmune overlap syndrome, 13 active bilharziasis and 10 Wilson’s disease. Minor number of cases was diagnosed by different other etiologies. Initial diagnosis was made by liver biopsy and confirmed by clinical response and laboratory findings. Liver biopsy is still considered as the gold standard diagnostic measure of different liver diseases representing an integral component of management decisions in hepatology.

## Introduction

There is no doubt that histopathological examination of liver biopsy (LB) is valuable in providing informative data about liver tissue with proper evaluation of the degree of hepatic injury and fibrosis [1]. The integration of clinical and laboratory data in conjunction with liver histological examination aid the clinicians toward better evaluation of liver diseases [2]. Despite the invasiveness, cost, reported complications, along with the inter- and intra-observer misconceptions, LB had kept its role as a sole final key for most unresolved liver disorders [3]. In the interferon era, LB was mandatory for hepatitis C virus (HCV) treatment decisions [4]. Nowadays, with the emerging generations of direct acting antiviral drugs (DAAs) against HCV, LB is claimed to be limitedly used [5]. Additionally, in advanced diseases, development of liver cirrhosis might veil the original causative pathology sufficing with the clinical, laboratory and radiological data [6]. Moreover, the ongoing impressive development of noninvasive radiological measures estimating hepatic fibrosis had weakened the need of LB [7]. All these novelties together might pose an owed question about the rationale of performing LB. Accordingly, reevaluation of the role LB as an undying effectual diagnostic approach in the era of DAAs was the impulse of designing this study.

## Patients and methods

### Patients’ selection

This observational longitudinal retrospective study was conducted on patients who had undergone LB at the National liver Institute (NLI), Menoufia University, Egypt, from January 2015 through December 2018. Unexplained elevations of liver transaminases and or hepatic cholestasis, along with unexplained liver-related conditions were mostly the indications of LB. All patients were subjected to thorough history taking and detailed clinical examination. Complete liver panel tests including transaminases, bilirubin, albumin, alkaline phosphatase, gamma glutamyl transferase, international normalized ratio (INR), complete blood picture, serum creatinine, blood urea and alpha-fetoprotein. Viral markers such as HCV Ab, HBsAg, HBc IgM, HBcIgG were done to all cases. HIV Ab, EBV IgM, HSVIgM, HDV IgM were done only if needed.

Other laboratory investigations aiming at helping diagnosis were done only when indicated like serum total IgG level, ANA, ASMA and LKM-1, AMA anti-neutrophil cytoplasmic (ANCA) and anti-double-strand DNA, angiotensin converting enzyme, copper and ceruloplasmin, and 24 h urinary copper. HCV-RNA was performed to all HCV infected patients using real-time PCR technique using Roche COBAS AmpliPrep/COBAS TaqMan HCV assay (Roche Diagnostics, Germany). HBV-DNA levels were obtained for all HBV infected patients using real-time PCR technique using Roche COBAS AmpliPrep/COBAS TaqMan HBV Test (Roche Diagnostics, Germany). The protocol was approved by the Institutional Review Board of the National Liver Institute, Menoufia University. This study conforms to the ethical guidelines of the 1975 Declaration of Helsinki.

### Radiologically

Abdominal ultrasound and Doppler on portal and hepatic veins with special emphasis on determining liver echogenicity, portal vein patency, spleen size and fluid collection and renal echogenicity were performed. Other radiological investigations data were done if needed like triphasic spiral CT or Magnetic resonance imaging (MRI) on the abdomen, Magnetic resonance pancreatography (MRCP), chest X-ray.

### Liver biopsy (LB)

Percutaneous ultrasound or CT-guided LB was taken (core not less than 1 cm, taken intercostally from right lobe of the liver) under both local (xylocaine) and light sedation (midazolam 2–3 mg intravenously). A written informed consent was obtained from all cases or their relatives. Patients on antiplatelet drugs and anticoagulants stopped taking these medications one week before LB. The patient sited in the supine position with the right hand placed under the head, and venous access was established in the left arm. The routine use of ultrasound was helpful in establishing the best location for biopsy, visualizing the liver during inspiration and expiration and identifying neighboring organs (i.e., lung, kidney, gut) that may be at risk of inadvertent puncture. The location for biopsy was marked clearly, and then, the area was prepped and draped in a sterile fashion [8]. One percent lidocaine (xylocaine) was injected over the upper border of a rib avoiding the intercostal nerve and vessels that run along the lower border. A small incision was made, and the patient held his or her breath when cutting needle was applied to obtain biopsy using either the manual Tru-Cut needle or the spring-loaded automatic needles. LB was fixed in formalin and embedded in paraffin. The patient was kept under observation for at 6 h and then discharged if no complications were reported.

### Histopathological examination

LB was subjected to routine processing for histopathological examination. All tissue sections were stained with hematoxylin and eosin, masson trichrome, orcein and Perls’ stain. Liver tissues that revealed biliary injury, bile ducts and ductules were visualized by CK7 immunostaining. Two experienced liver histopathologists evaluated the histological changes in liver sections unaware of the clinical diagnosis.

The exclusion criteria in these series were patients with extrahepatic biliary obstruction, hydatid cyst, ascites and those with severe coagulopathy.

### Statistical analysis

Data were collected, tabulated and statistically analyzed using an IBM personal computer with Statistical Package of Social Science (SPSS) version 20 where the following statistics were applied. Descriptive statistics, in which quantitative data were presented in the form of mean ($\overline{X}$), standard deviation (SD), range and qualitative data, had been presented in the form of numbers and percentages.

## Results

Patients’ characteristics: A total of 275 liver biopsies were collected, 191 males and 84 females with mean age 41.22 ± 13.36.

Different etiological diagnosis were obtained from histopathological examination and were classified as shown in Table 1: DILI comprising 48/275(17.45%) cases followed by biliary cholangitis 47 (17.09%) (primary sclerosing cholangitis, primary biliary cholangitis), 42 (15.27%) non-alcoholic fatty liver disease (NAFLD), 36 (13.09%) autoimmune hepatic liver disease (autoimmune hepatitis (AIH), autoimmune overlap), 34 (12.36%) chronic viral hepatitis infection (HCV and HBV) and 11 (4%) sarcoidosis. Thirteen cases with active bilharziasis were responsible for 4.72% of all indications for LB.

In the current study, the major indications for LB were DILI and were responsible for 17.45% of all indications of LB in our series. Although LB was not indicated in DILI cases proved clinically, the main indication of LB was in atypical cases or cases denied drug history. LB had established the diagnosis of DILI in patients taking a common prescribed drug, herbals or pesticides exposure. The reported pathological features were either combined hepatitis and cholestasis 21.5%, chronic hepatitis in 21.5%, intrahepatic cholestasis 21.5%, combined cholestasis and steatosis in 10.6%, acute hepatitis 5.3%, syncytial hepatitis 5.3% or steatohepatitis in 5.3%. Eosinophils-rich infiltrate was a nearly constant finding in most of cases.

Table 2 showed most common drugs encountered in DILI. It was evident in our study group that nonsteroidal anti-inflammatory drug diclofenac was the most common cause of DILI followed by herbal medicine, amoxicillin–clavulanic acid, halothane anesthetic agent, amiodarone, pesticides and disinfectants. Rare cases of DILI necessitating LB were isoniazid, atorvastatin, estrogen and carbimazole.

**Table 1:** Indications of liver biopsy at the National Liver Institute, Egypt

Indications for LB	N (%)
DILI	48 (17.45)
Biliary diseases	47 (17.09)
PSC	34 (12.36)
PBC	13 (4.72)
NAFLD	42 (15.27)
Autoimmune liver disease	36 (13.09)
AIH	29 (10.54)
Autoimmune overlap syndrome	7 (2.54)
Chronic viral hepatitis	34 (12.36)
Chronic HBV	25 (9.09)
Chronic HCV	5 (1.81)
Dual HCV and HBV infection	4 (1.45)
Sarcoidosis	11 (4)
Active bilharziasis	13 (4.72)
Wilson's disease	10 (3.63)
Non-hepatotropic viruses	10 (3.63)
CMV	6 (2.18)
EBV	4 (1.45)
Dubin–Johnson syndrome	7 (2.54)
Liver abscess	6 (2.18)
Resolving acute hepatitis	4 (1.45)
Rare cases	7 (2.54)
Latent congenital hepatic fibrosis	1 (0.36)
Amyloidosis	
Hemochromatosis	1 (0.36)
Hepatic amoebiasis	1 (0.36)
Malaria	1 (0.36)
Polyarteritis nodosa	1 (0.36)
Myeloproliferative disease	1 (0.36)
	1 (0.36)

DILI drug-induced liver injury, NAFLD non-alcoholic fatty liver disease, HCV hepatitis C virus, HBV hepatitis B virus, AIH autoimmune hepatitis, PSC primary sclerosing cholangitis, PBC primary biliary cholangitis, CMV cytomegalovirus, EBV Epstein–Barr virus

**Table 2:** Most implicated drugs in drug-induced liver injury in the present study

Drugs (liver biopsy in only 48 cases)	N (%)
Nonsteroidal anti-inflammatory drug	10 (20.3)
Herbal remedies	8 (16.6)
Amoxicillin–clavulanic acid	5 (10.4)
Halothane anesthesia	5 (10.4)
Unrecognized type of drug intake	5 (10.4)
Amiodarone	4 (8.3)
Pesticides and disinfectants	4 (8.3)
Oral contraceptives	2 (4.1)
Isoniazid	2 (4.1)
Lipid-lowering agent atorvastatin	2 (4.1)
Carbimazole	1 (2.08)

LB played an initial diagnostic role in five cases of early-stage biliary disease (10.6% of biliary cholangitis cases) on which the patient presented by elevated liver enzymes with normal or mild elevated bilirubin level. In addition, in AIH cases, LB had a key role in establishing the diagnosis in three cases (8.3% of AIH cases) presenting with negative autoantibodies and in raising the possibility of associated biliary overlap disease in six cases (16.7% of AIH cases) apart from defining the grade and stage of disease.

In NAFLD cases, LB confirmed the clinical diagnosis and differentiated NAFLD from NASH in 35 cases (83.3% of NAFLD cases). However, in the remaining 7 cases (16.7% of NAFLD cases), the patients were slim and had no diabetes or insulin resistance and normal abdominal sonography. The liver pathology showed a mild steatosis (20%), ballooning or lobular inflammation which raised the diagnosis of lean NAFLD.

LB remained the gold standard in diagnosis of granulomatous liver disease and defining the causative agents (bilharzial ova in 13 cases and numerable unpaired granulomas suspicious for sarcoidosis in 11 cases).

There were 10 cases diagnosed as Wilson disease. Table 3 showed the clinical and laboratory data of those cases.

**Table 3:** Clinical and laboratory data of Wilson cases

	Wilson cases
	N = 10
Age (years)	
Mean ± SD	27.90 ± 12.28
Median (range)	22.5 (17–55)
Sex	
Male	3 (30.0)
Female	7 (70.0)
AST (U/L)	
Mean ± SD	106.70 ± 69.80
Median (range)	81 (65–300)
ALT (U/L)	
Mean ± SD	117.80 ± 106.36
Median (range)	79.5 (46–400)
Alkaline phosphatase (U/L)	
Mean ± SD	115.70 ± 48.08
Median (range)	100 (76–232)
PC %	
Mean ± SD	78.79 ± 7.68
Median (range)	80 (63.9–90)
International normalized ratio (INR)	
Mean ± SD	1.22 ± 0.08
Median (range)	1.20 (1.1–1.39)
Total bilirubin (mg/dl)	
Mean ± SD	2.95 ± 2.24
Median (range)	3 (0.51–7.9)
D. bilirubin (mg/dl)	
Mean ± SD	2.16 ± 1.85
Median (range)	2.3 (0.12–6)
Albumin (g/dl)	
Mean ± SD	3.67 ± 0.87
Median (range)	3.9 (1.6–4.8)
Hemoglobin	
Mean ± SD (g/dl)	11.70 ± 1.14
Median (range)	11.2 (10.1–13.4)
WBCs × 10^3^	
Mean ± SD	5.54 ± 1.47
Median (range)	5.55 (3.2–7.6)
Platelets × 10^3^	
Mean ± SD	153.90 ± 52.31
Median (range)	156 (60–231)
**Abdominal ultrasound**	
*Liver*	
Non-cirrhotic	4(40.0)
Cirrhotic	6 (60.0)
Splenomegaly	
No	5 (50.0)
Yes	5 (50.0)

**Fig. 1:**
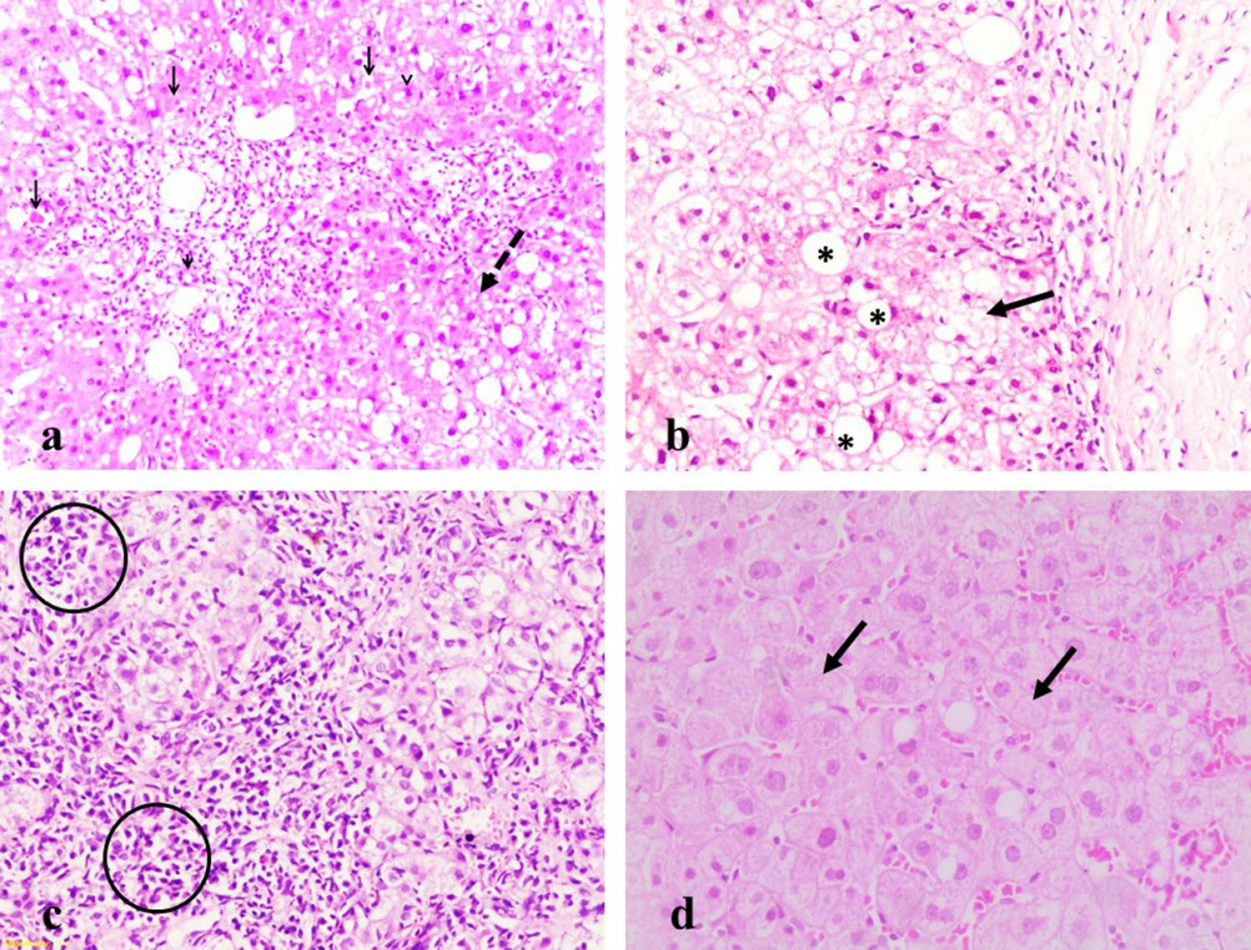
Histopatholoigal findings in DILI, primary sclerosing cholangitis, primary biliary cholangitis and acute hepatitis

Figure 1 demonstrates the most frequent etiological diagnosis made by LB histopathological evaluation. Liver tissue examination from cases of DILI exhibited microvesicular steatosis, frequent apoptotic bodies, cholestatic rosettes and inflammatory infiltrate rich in eosinophils in portal tracts (Fig. 1a). Primary sclerosing cholangitis and primary biliary cholangitis were clearly visualized by masson trichrome stain and CK7 immunostaining of destructive bile ducts and ductules and showed bile ductular proliferation and bilirubin stasis (Fig. 1b, c). Liver sections exhibited prominent plasmalymphocytic infiltrate together with plasma cell clusters were consistent with autoimmune hepatitis (Fig. 1d). Autoimmune overlap syndrome displayed combined hepatitis and biliary injury as shown in Fig. 1. For HCV patients who did not receive DAAs and had developed elevated liver enzymes, their LB revealed features of chronic liver disease with variable stage of fibrosis (Fig. 1).

**Fig. 2:**
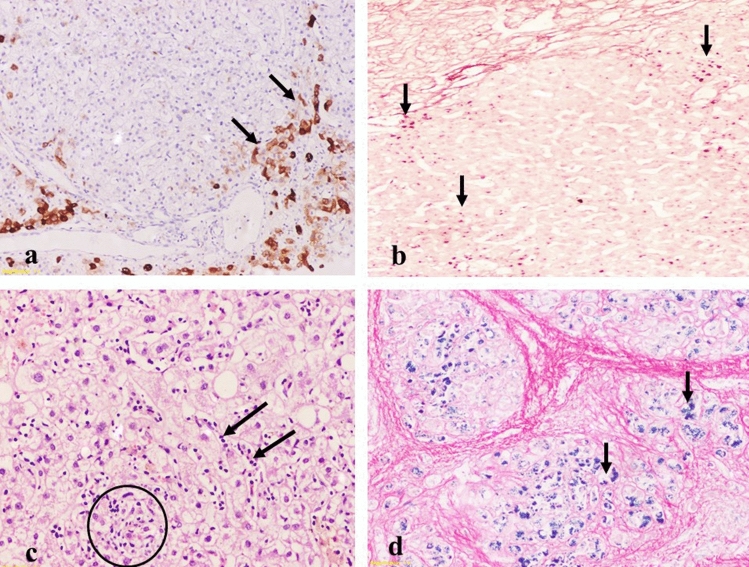
Histopatholoigal findings in Non-hepatotropic viruses (Epstein-Barr or cytomegaloviruses),Wilson's disease, Dubbin–Johnson syndrome and resolving acute hepatitis

Figure 2 shows less common diagnosis made by LB examination. Non-hepatotropic viruses (Epstein–Barr or cytomegaloviruses) exhibited sinusoidal beaded lymphocytes, viral inclusions and microabcesses (Fig. 2a). Wilson's disease was identified histologically by orcein stain-positive copper-associated protein deposits (Fig. 2b). Indications for LB in these cases were 10 (3.63%) cases for each. Seven cases were diagnosed as Dubin–Johnson syndrome as the LB examination revealed preserved architecture and normal looking hepatocytes except for perivenular zone exhibited bilirubin stasis in the cytoplasm of hepatocytes (Fig. 2c). Six cases were diagnosed as liver abscess. Four cases were diagnosed as resolving acute hepatitis that were identified by the hypertrophied Kupffer cells, frequent apoptotic bodies and resetting of hepatocytes (Fig. 2d). Very rare cases presented as a solitary case each like latent congenital hepatic fibrosis (Fig. 2), amyloidosis, hemochromatosis (Fig. 2), hepatic amoebiasis, malaria, polyarteritis nodosa, myeloproliferative disease.

The reported complications after LB in the current study are illustrated in Table 4. Pain was the most evident minor complications and was present in 93.09% patients. Analgesic agent was required in 71/275(25.18%) of cases to control pain. Major complications were rare and subsequently seen in 5 (1.8%) cases with no death. Bleeding occurred in 2 cases and one case was severe requiring blood transfusion and conservative treatment was enough. The other complications were one case (0.36%) for either biliary leak, pneumothorax or sepsis.

**Table 4:** Complications of liver biopsy

Complications of liver biopsy	N (%)
*Minor complications*	
Pain requiring analgesia	71 (25.18)
Major complications	5 (1.8)
Bleeding	2 (0.72)
Massive requiring blood transfusions	1 (0.36)
Minimal (conservative treatment)	1 (0.36)
Biliary leak	1 (0.36)
Sepsis	1 (0.36)
Pneumothorax	1 (0.36)
Death	0

## Discussion

Since the advent of DAAs, the number of daily done liver biopsies (LB) had been considerably lessened [9]. Additionally, the suggested high accuracy of the noninvasive measures had imposed a big query on the current significance of LB in liver disease diagnosis. Confidently, LB is never to be outdated, and there will be always a substantial role for histopathological examination in solving diagnostic dilemmas [8]. In this unique study, we had investigated the current diagnostic needs of LB after quitting interferon therapy which was mainly dependent on LB finding adoption of the new DAAs.

Drug-induced liver injury (DILI) is one of the most common indications of LB as its diagnosis is mainly based on exclusion of all other etiologies of liver diseases which is practically impossible without the print of LB [10].

Accordingly, DILI had attained the highest number of cases that had performed LB and included in the current study (17.45%). The presence of combined hepatitis and cholestasis, and eosinophilia, along with centrilobular necrosis, is the most prominent features of DILI. However, a wide range of histopathological patterns had been identified in DILI cases according to the offending drug. Picture of acute hepatitis, acute liver failure, steatohepatitis, granulomatous hepatitis and eventually some cases develop chronic hepatitis leading to liver cirrhosis [11]. Once cirrhosis had been developed, the recognition of the primary pathology is significantly concealed. In a recent Egyptian study, nonsteroidal anti-inflammatory (NSAID) drugs was reported as the first offending drug in DILI occurrence [10]. This finding was similar to the results of the current study, where NSAID represented about 20% of cases, with 8% for herbal preparations, and 5% each of amoxycillin clavulanic acid and halothane.

Unrecognized drugs also represent 5% of cases which is a threatening alarm for more social orientation of drug hazards along with more governmental censorship for limitation of over the counter drugs.

NAFLD is considered the evolving most common cause of liver diseases nowadays. In the current study, NAFLD was the second prevalent indication of LB 42(12.75%). The global prevalence of NAFLD in the Middle East is high and affects about 31.8% of adult population [12]**.** Based on the strong association between NAFLD and the metabolic syndrome, a new nomination had been emerged to be metabolism-associated fatty liver disease (MAFLD) [13]. Hepatic steatosis more than 5% along with ballooning, lobular inflammation and even fibrosis are the main characteristics of NASH [14]. Despite the availability of many noninvasive measures for evaluation of steatosis and specifically fibrosis, LB is still the most accurate distinguishing maneuver of NASH from NAFLD [15]**.** In MAFLD, the linkage between liver fibrosis and cardiovascular risks is well identified denoting higher rates of morbidity and mortality [13]. Accordingly, LB is still mandated in this area as either a diagnostic or a prognostic measure. In spite of the necessity of LB in NAFLD diagnosis in all international guidelines, the only obstacle prohibiting generalization of this concept is the patient’s refusal. More efforts should be wielded to delineate and eliminate all patients’ fairs and illusions about LB.

In the current study, chronic viral hepatitis infections were the fourth cause of performing LB (12.36%). Five cases with hepatitis C, 25 cases with hepatitis B virus infection and 4 cases with dual hepatitis B and C infection.

The unprecedented Egyptian campaign for treating HCV was greatly appreciated all over the world. Since the emergence of direct acting antiviral drugs (DAAs) against HCV, the number of performed LBs had remarkably lessened. Unlike interferon therapy of HCV, DAAs had limited the reliance on LB as a prerequisite of treatment decisions [7]**.** However, LB is still indicated in chronic HCV cases that showed poor sustained viral response in order to define a concomitant disease either iron overload, steatosis or granuloma that could resist the therapy.

In chronic active hepatitis B virus (HBV) infection, antiviral therapy can often be initiated without the need of LB. However, the difficulty in determining disease activity and fibrosis by serologic and biochemical data in chronic HBV makes LB an important tool in the management of a subset of patients [7]**.** Demonstration of the degree of fibrosis and/or inflammation or associated concomitant disease in LB is a decisive factor for both treatment indications [16]**.**

In patients who have HBV-DNA 2,000 IU/ml and at least moderate fibrosis, treatment may be initiated even if ALT levels are normal [17]**.** Moreover, the debatable queries about either to treat or not those with HBV chronic active infections could be partially resolved relying on histopathological examination of LB [18]**.**

The treatment of dual HCV and HBV infections are challenging, as treating HCV with DAAs had been associated with increase in HBV-DNA levels in patients with positive HBsAg. In some cases, elevated ALT and even liver failure may develop in these conditions [19, 20]**.** Accordingly, LB might be of significance in determining the most prevailing infection and determining treatment priorities.

In our series, AIH patients are representing about 13% of the whole cohort. From our data, LB is the most trustworthy diagnostic measure of AIH included in all old and recent scoring systems of AIH recommended by international guidelines [21]. Remission of AIH is considered in those who develop normalization of both serum transaminases and IgG [22]. However, normalization of either of them mandates reliance on histopathological examination of LB to assure remission [21]. LB should be performed also for immunosuppression resistant cases for the possibility of overlap syndrome (which was detected in 2.45% of cases in the current study). Recognition of the prevailing pathology in overlap syndrome could not be assured without LB. Nevertheless, progression to liver cirrhosis in those patients remains the obstacle masquerading the prime pathology [21].

In the current series, biliary tract diseases especially biliary cholangitis (PSC, PBC) are considered important causes of liver disease. They represent 17.9% of liver biopsies in the National liver disease. Biliary diseases should be taken in consideration as an important cause of liver disease as prolonged biliary inflammation leads to many serious complications like cholangitis, biliary abscesses and liver cirrhosis which is a fast devastating incident in this context [23]. Biliary cirrhosis is distinguished to have a hastier course rather than other liver pathologies, a fact mandating early diagnosis and rapid management [24]. Unlike PBC, LB is not mandated for most of PSC cases as magnetic resonance cholangiography (MRCP) and/or endoscopic retrograde cholangiography (ERCP) remains the most reliable diagnostic measure [25]. Cases with small duct PSC or overlap syndromes with PSC were the only cases relying on LB results [26]. While destruction of small bile ducts is the most pathognomonic histopathological feature of PBC, the presence of onion skin fibrosis is the devastating sign of PSC [27].

Granulomatous hepatitis is the most diagnostic feature of sarcoidosis representing 4% of the current study cases. Diagnosis of cases with hepatic sarcoidosis might be challenging for the non-specific presentations. LB might be the last resolution for this dilemma. Non-caseating numerable unpaired granuloma and intrahepatic cholestasis were found to be the prevailing histological features of hepatic sarcoidosis [28]. However, sometimes the distinction from early non-caseating TB granuloma is frustrating necessitating resorting to more distinctive special stains [28].

The need of LB for recognition of hepatic schistosomiasis was reported in 4.72% of current study cases. Schistosomal non-cirrhotic portal hypertension was the most prevalent diagnostic need for LB with the characteristic hepatic granuloma [29]. Thanks to the governmental efforts, the prevalence of hepatic schistosomiasis had been dramatically ameliorated in Egypt [30].

Long ago, LB is only needed for exclusion of other causes of liver disease rather than diagnosis of Wilson disease (WD). The required dried weight liver tissue for proper diagnosis of WD is a cumbersome against LB. The wide range of histopathological presentations might also be another con [31]. In accord, LB was needed in only in 10 cases (3.63%) in this research that were with clinical and laboratory inconclusive data.

Despite rarity, liver might be the seat of non-hepatotropic viral infections. Sometimes LB might be the leading suggestive measure of non-hepatotropic viral infections while searching of cause of the liver injury in a puzzling case [32]. Epstein–Barr virus (EBV) had affected 6 cases while cytomegalovirus (CMV) had affected 4 cases of patients included in the current study. The presence of lobular disarray, nuclear inclusion bodies, microabscesses and lobular lymphocytic infiltration are the main histopathological features suggestive of CMV in LB [32].

In EBV, portal tracts are massively intruded by atypical lymphocytes and plasma cells. Intra-sinusoidal lymphocytosis often has a characteristic “Indian-file” appearance [32].

Seven cases of Dubin–Johnson syndrome were reported in the current study. Surprisingly these cases might present up to fifties with unexplained conjugated hyperbilirubinemia and normal transaminases. Grossly the liver appeared black with the absence of anti-MRP2 on immunohistochemical staining of liver tissue [33]**.**

Liver abscess is a rare indications for liver biopsy in our study and represents less than 2%. In cryptogenic pyogenic liver abscess is present in 60% of cases whenever there is no obvious identifiable risk factor [34]**.** Most pyogenic liver abscesses are located in the right lobe of the liver because of portal vein anatomy, more hepatic mass and dense bile canaliculi in this lobe [35]**.** This is also prominent in our series as 5 of 6 liver abscesses were found to be located in the right lobe of the liver. The imaging of the liver plays a corner stone in helping detection of liver abscess. Abdominal ultrasound and CT scan can help in diagnosis of 50% of cases [36]**.** Nevertheless, LB is requested only in cases of early hepatic masses before evident liquefaction along with atypical imaging criteria.

Solitary cases of latent congenital hepatic fibrosis, amyloidosis, hemochromatosis, hepatic amoebiasis, malaria, polyarteritis nodosa, myeloproliferative disease were also reported in the current series.

The imaging-guided LB had limited the associated risks of this interventional theoretically invasive maneuver. Regarding LB complications in the current study, pain requiring analgesia was the main complain (25.18%). Major complications represented only less than 1.18% with no reported mortalities. Bleeding (0.7%), biliary leak (03%), sepsis (0.03%) and pneumothorax (0.3%) were reported without either surgical interventions or unfavorable outcomes. Similarly, Elsenberg and his colleagues had denoted the occurrence of pain even mild discomfort following LB in 84% [37]**.** Also, Kose et al. had reported that post-LB pain requiring analgesia was present in 19.8% and major complications in 1.15%. Major complications in their study included pneumothorax in 0.17%, haemobilia in 0.08% and hematoma in 0.9%) and absence of other complications like abscess, bacteremia, organ perforation and death [38]**.** Also, Govender et al. reported serious complications in a rate of 1.7% in their series in the form of pneumothorax, symptomatic hematoma and pseudoaneurysm and no deaths [39]**.** The mortality rate attributed to the procedure is estimated in one per 10 thousand biopsies and is usually secondary to bleeding; this is even lower when the biopsy is not performed for evaluation of liver tumors [40]**.** The reported higher rates of safety had denied any claims of high-risk probabilities.

In the current study, the complete absence of what was known as intra-observer variations denoting the highly professional skillful pathology team, adding more reliability to the maneuver.

## Conclusion

The tapered decline in LB requests upon the advent of DAAs treating HCV infections along with the higher rates of accuracy of laboratory and imaging diagnostic measures had been questioned lately. However, LB will remain the golden key of lots of hepatic diagnostic dilemmas. Additionally, the higher reported safety rates might help in gaining more confidence among refusal parties. Currently, the mapping of liver diseases prevalence had been significantly changed. While MAFLD, DILI and AIH are the main indications of LB nowadays, the need of LB in viral infections had become substantially limited.

## References

[1] Tapper EB, Lok AS (2017). Use of liver imaging and biopsy in clinical practice. N Engl J Med.

[2] Ovchinsky N, Moreira RK, Lefkowitch JH, Lavine JE (2012). Liver biopsy in modern clinical practice: a pediatric point-of-view. Adv Anat Pathol.

[3] Pandey N, Hoilat GJ, John S. Liver Biopsy. [Updated 2020 Dec 9]. In: StatPearls [Internet]. Treasure Island (FL): StatPearls Publishing; 2020 Jan. https://www.ncbi.nlm.nih.gov/books/NBK470567/.

[4] Sherman M, Shafran S, Burak K, Doucette K, Wong W, Girgrah N, Yoshida E, Renner E, Wong P, Deschênes M. Management of chronic hepatitis C: consensus guidelines. Can J Gastroenterol. 2007;21 Suppl C (Suppl C):25C-34C. PMC2794457 17568824

[5] Gonzalez HC, Jafri SM, Gordon SC (2013). Role of liver biopsy in the era of direct-acting antivirals. Curr Gastroenterol Rep.

[6] Bosman FT, Carneiro F, Hruban RH, Theise ND (2010) Hepatocellular carcinoma. WHO Classification of tumours of the Digestive System, 4th Edition, International Agency for Research on Cancer. Lyon. 2010: 348–402.

[7] Misdraji J (2014). Changing indications for liver biopsy: viral hepatitis. Diagn Histopathol.

[8] Boyd A, Cain O, Chauhan A, Jame G, Webb GJ (2020). Medical liver biopsy: background, indications, procedure and histopathology. Front Gastroenterol.

[9] Mansour RH, Zaky S, El Kassas M, Mamdouh H, Esmat G (2019). Evaluating the effect of direct-acting agents on liver fibrosis, by real-time elastography, Fibroscan and FIB4 score in chronic HCV patients. Sci J Al-Azhar Med Fac Girls.

[10] Alhaddad O, Elsabaawy M, Abdelsameea E (2020). Presentations, causes and outcomes of drug-induced liver injury in Egypt. Sci Rep.

[11] Björnsson ES, Bergmann OM, Björnsson HK, Kvaran RB, Sigurdur O (2013). Incidence, presentation, and outcomes in patients with drug-induced liver injury in the general population of Iceland. Gastroenterology.

[12] Younossi ZM, Marchesini G, Pinto-Cortez H, Petta S (2019). Epidemiology of nonalcoholic fatty liver disease and nonalcoholic steatohepatitis: implications for liver transplantation. Transplantation.

[13] Fouad Y, Waked I, Bollipo S, Gomaa A, Ajlouni Y, Attia D (2020). What's in a name? Renaming ‘NAFLD’ to ‘MAFLD’. Liver Int.

[14] Chalasani N, Younossi Z, Lavine JE (2018). The diagnosis and management of nonalcoholic fatty liver disease: Practice guidance from the American Association for the Study of Liver Diseases. Hepatology.

[15] Yeh MM, Brunt EM (2014). Pathological features of fatty liver disease. Gastroenterology.

[16] Gameel KH, M. Elsabaawy M, Naguib M, et al. Role of liver biopsy in HBV infected egyptian patients: a new insight. Br J Med Med Res, ISSN: 2231-0614: 19, 2

[17] EASL 2017 Clinical Practice Guidelines on the management of hepatitis B virus infection. J Hepatol. 2017;67(2):370–398. 10.1016/j.jhep.2017.03.021 28427875

[18] Metwally K, Elsabaawy M, Abdel-Samiee M, Morad W, Ehsan N, Abdelsameea E (2020). FIB-5 versus FIB-4 index for assessment of hepatic fibrosis in chronic hepatitis B affected patients. Clin Exp Hepatol.

[19] Bersoff-Matcha SJ, Cao K, Jason M, Ajao A, Jones SC, MeyerT, Brinker A. Hepatitis B virus reactivation associated with direct-acting antiviral therapy for chronic hepatitis C Virus: a review of cases reported to the US food and drug administration adverse event reporting system. Ann Intern Med 2017; 166: 792–798. 10.7326/M17-0377 28437794

[20] Belperio PS, Shahoumian TA, Mole LA, Backus LI (2017). Evaluation of hepatitis B reactivation among 62,920 veterans treated with oral hepatitis C antivirals. Hepatology.

[21] Mack CL, Adams D, Assis DN (2020). Diagnosis and management of autoimmune hepatitis in adults and children: 2019 practice guidance and guidelines from the American association for the study of liver diseases. Hepatology.

[22] Tiniakos DG, Brain JG, Bury YA Tiniakos. Role of Histopathology in Autoimmune Hepatitis. Dig Dis. 2015;33 Suppl 2:53–64. 10.1159/000440747 26642062

[23] Lindor KD, Bowlus CL, Boyer J, Levy C, Mayo M (2019). primary biliary cholangitis: 2018 practice guidance from the American association for the study of liver diseases. Hepatology.

[24] Purohit T, Cappell MS (2015). Primary biliary cirrhosis: Pathophysiology, clinical presentation and therapy. World J Hepatol.

[25] Sirpal S, Chandok N (2017). Primary sclerosing cholangitis: diagnostic and management challenges. Clin Exp Gastroenterol.

[26] Igarashi G, Endo T, Mikami K (2017). two cases of primary sclerosing cholangitis overlapping with autoimmune hepatitis in adults. Intern Med.

[27] Tabibian JH, Bowlus CL (2017). Primary sclerosing cholangitis: a review and update. Liver Res.

[28] Doppalapudi H, Markus JT, Parekh U. Granulomatous Hepatitis. [Updated 2020 Nov 3]. In: StatPearls [Internet]. Treasure Island (FL): StatPearls Publishing; 2020 Jan-. Available from: https://www.ncbi.nlm.nih.gov/books/NBK564315/33231985

[29] Parris V, Michie K, Andrews T, et al. Schistosomiasis japonicum diagnosed on liver biopsy in a patient with hepatitis B co-infection: a case report. J Med Case Rep. 2014; 10.1186/1752-1947-8-45. 10.1186/1752-1947-8-45 PMC3930008 24521427

[30] Rollinson D, Knopp S, Levitz S, Stothard JR, Tchuem Tchuenté LA (2013). Time to set the agenda for schistosomiasis elimination. Acta Trop.

[31] Oe S, Honma Y, Yabuki K (2020). Importance of a liver biopsy in the management of Wilson disease. Intern Med.

[32] Dancygier H. Viral infections by nonhepatotropic viruses. Clin Hepatol. 2010;823–830.

[33] Devgun MS, El-Nujumi AM, O’Dowd GJ, Barbu V, Poupon R (2012). Noval mutations in the Dubin-Johnson syndrome gene ABCC2/MRP2 and associated biochemical changes. Ann Clin Biochem.

[34] Malik AA, Bari SU, Rouf KA (2010). Pyogenic liver abscess: changing patterns in approach. World J Gastrointest Surg.

[35] Serraino C, Elia C, Bracco C (2018). Characteristics and management of pyogenic liver abscess: A European experience. Medicine (Baltimore).

[36] Lo JZ, Leow JJ, Ng PL (2015). Predictors of therapy failure in a series of 741 adult pyogenic liver abscesses. J Hepatobiliary Pancreat Sci.

[37] Eisenberg E, Konopniki M, Veitsman E, Kramskay R, Gaitini D, BaruchY. Prevalence and characteristics of pain induced by percutaneous liver biopsy. Anesth Analg 2003; 96:1392-1396 10.1213/01.ANE.0000060453.74744.17 12707140

[38] Kose S, Ersan G, Tatar B, Adar P, Sengel BE (2015). Evaluation of percutaneous liver biopsy complications in patients with chronic viral hepatitis. Eurasian J Med.

[39] Govender P, Jonas MM, Alomari AI (2013). Sonography-guided percutaneous liver biopsies in children. AJR J Roentgenol.

[40] West J, Card TR (2010). Reduced mortality rates following elective percutaneous liver biopsies. Gastroenterology.

